# Physiology dictated treatment after severe trauma: timing is everything

**DOI:** 10.1007/s00068-022-01916-z

**Published:** 2022-02-26

**Authors:** Karlijn J. P. van Wessem, Luke P. H. Leenen, Falco Hietbrink

**Affiliations:** Department of Trauma Surgery, University Medical Center Utrecht, Heidelberglaan 100, 3584 CX Utrecht, The Netherlands

**Keywords:** Damage control surgery, Early total care, Outcome

## Abstract

**Introduction:**

Damage control strategies in resuscitation and (fracture) surgery have become standard of care in the treatment of severely injured patients. It is suggested that damage control improves survival and decreases the incidence of organ failure. However, these strategies can possibly increase the risk of complications such as infections. Indication for damage control procedures is guided by physiological parameters, type of injury, and the surgeon’s experience. We analyzed outcomes of severely injured patients who underwent emergency surgery.

**Methods:**

Severely injured patients, admitted to a level-1 trauma center ICU from 2016 to 2020 who were in need of ventilator support and required immediate surgical intervention ( ≤24 h) were included. Demographics, treatment, and outcome parameters were analyzed.

**Results:**

Hundred ninety-five patients were identified with a median ISS of 33 (IQR 25–38). Ninety-seven patients underwent immediate definitive surgery (ETC group), while 98 patients were first treated according to damage control principles with abbreviated surgery (DCS group). Although ISS was similar in both groups, DCS patients were younger, suffered from more severe truncal injuries, were more frequently in shock with more severe acidosis and coagulopathy, and received more blood products. ETC patients with traumatic brain injury needed more often a craniotomy. Seventy-four percent of DCS patients received definitive surgery in the second surgical procedure. There was no difference in mortality, nor any other outcome including organ failure and infections.

**Conclusions:**

When in severely injured patients treatment is dictated by physiology into either early definitive surgery or damage control with multiple shorter procedures stretched over several days combined with aggressive resuscitation with blood products, outcome is comparable in terms of complications.

**Supplementary Information:**

The online version contains supplementary material available at 10.1007/s00068-022-01916-z.

## Introduction

Damage control surgery (DCS) has gained popularity since the 1990s, and is characterized by a brief initial operation used to rapidly control hemorrhage, air leak, and/or contamination, (temporarily) restore blood flow and long bone stabilization with one or several abbreviated interventions. After surgery, physiology is optimized in the Intensive Care Unit (ICU) before returning to the operating room (OR) for definitive surgery [1, 2]. Although DCS has been regarded as a breakthrough in trauma care, there are no strict selection criteria reported in the literature. Selection of patients is based on a general consensus with criteria based on anatomical location of the injury (including major intra-abdominal (vascular) injury), and physiological parameters (acidosis, coagulopathy and hypothermia) [3, 4]. However, there are several other reasons to choose for abbreviated surgery, for example in patients with associated severe traumatic brain injury (TBI). With a wide range of criteria it is not surprising there is a wide variation in the use of DCS between trauma centers [3, 5]. DCS has shown to improve survival in the most severely injured patients, nevertheless the procedure is associated with a relatively high incidence of (infectious) complications, and prolonged length of stay [3, 6–9]. Some authors have questioned liberal DCS and warned for the overuse of damage control surgery [7, 10]. This has swung the pendulum once again resulting in a philosophy of providing Early Appropriate Care (EAC) [11–13]. EAC defined as providing a plan of action based on continual reassessment and reaction to the response to injury and surgery is mainly used in the context of orthopedic trauma and basically suggests to fix the bones at an early stage unless physiology deteriorates and definitive fixation should be abandoned [11–13]. However, it remains important that DCS is performed in the correctly selected patients in whom the benefit of the procedure exceeds its expected negative consequences.

The aim of this study was to evaluate these decisions for damage control surgery in both truncal and orthopedic surgery, and investigate whether these choices were appropriate in correlation with the outcome of severely injured patients. We hypothesized that the decisions for DCS were appropriate, expressed in comparable outcome in patients who received damage control surgery and patients who had early total care.

## Materials and methods

A prospective population-based cohort study was undertaken to investigate outcomes in severely injured patients admitted to the Intensive Care Unit (ICU) of a major (Level-1) trauma center (University Medical Center Utrecht, the Netherlands). From January 2016 till December 2020, all consecutive polytrauma (ISS > 15) patients  ≥15 years of age who were admitted to the adult ICU and underwent urgent surgery ( ≤24 h after admission) were included. Details of the hospital and catchment area were previously described [14]. Patients who were dead on arrival in ED or died prior to arrival in ICU were excluded.

Patients with isolated TBI, asphyxiation, drowning and burns were excluded, because of potential different physiologic response to severe trauma and a significantly different mortality and morbidity profile [15, 16]. Isolated injury to the brain was defined as Abbreviated Injury Score (AIS) head  ≥3 and AIS  ≤2 in other regions.

All data were prospectively collected by authors KW and LL and included demographics, shock and resuscitation parameters. Administration of both crystalloid and blood products including Packed Red Blood Cells (PRBC), Fresh Frozen Plasma (FFP) and Platelets (PLT) was documented in the first 24 h after admission. Further, detailed data of five initial surgical interventions per patient within the first 10 days after admission were documented and contained type, timing and duration of surgery (total time in OR), per-operative physiology (base deficit, hemoglobin, temperature) and resuscitation parameters (crystalloids, PRBC, FFP, PLT, tranexamic acid (TXA)). Additionally, Denver MOF scores [17], and ARDS Berlin criteria [18] were registered daily up until 28 days or discharge from ICU.

Damage control surgery was defined as any surgery (both truncal and orthopedic) that was abbreviated to restore normal physiology before returning to OR for definitive treatment.

In our hospital, the selection for damage control surgery is in correlation with the general literature consensus [19, 20], and based on a combination of physiological parameters (acidosis (base deficit  ≤-6.0 mmol/L), hypothermia (temperature  ≤34 °C), coagulopathy (Prothrombin Time (PT)  ≥16 s), anatomical locations of the injuries, associated injuries, patient’s response to the given care, and surgeon’s discretion. Patients who initially underwent DCS often needed additional surgeries during their hospital stay (fracture fixation, repeated debridement for soft tissue injuries, mesh approximation in open abdomen, etc.). These surgeries were regarded as (ongoing) definitive surgical care (DS).

Early total care (ETC) was defined as definitive fixation of fractures, and/or definitive treatment of injuries in chest and abdomen in the early phase after injury ( ≤24 h). Patients who had definitive surgery in multiple procedures stretched over several days were also included in ETC group.

Primary outcome was to evaluate the decision to perform either DCS or ETC in severely injured patients, and investigate whether these choices were appropriate in correlation with in-hospital mortality. Secondary outcome was the correlation between both types of treatment and adverse outcomes during hospital stay such as MODS, ARDS, thrombo-embolic and infectious complications.

### Ethical approval

The local ethics committee approved this prospective observational study and waived consent (reference number WAG/mb/16/026664).

### Statistical analysis

All statistical analysis were performed using IBM SPSS Statistics, version 26.0 (Armonk, NY, USA). Results are presented as median and interquartile range (IQR). Kruksal-Wallis was used to test continuous variables for equality, whereas Chi-Square or Fisher’s exact test (values less than 6) were used to test categorical data. Statistical significance was set at *P* < 0.05.

## Results

**Fig. 1:**
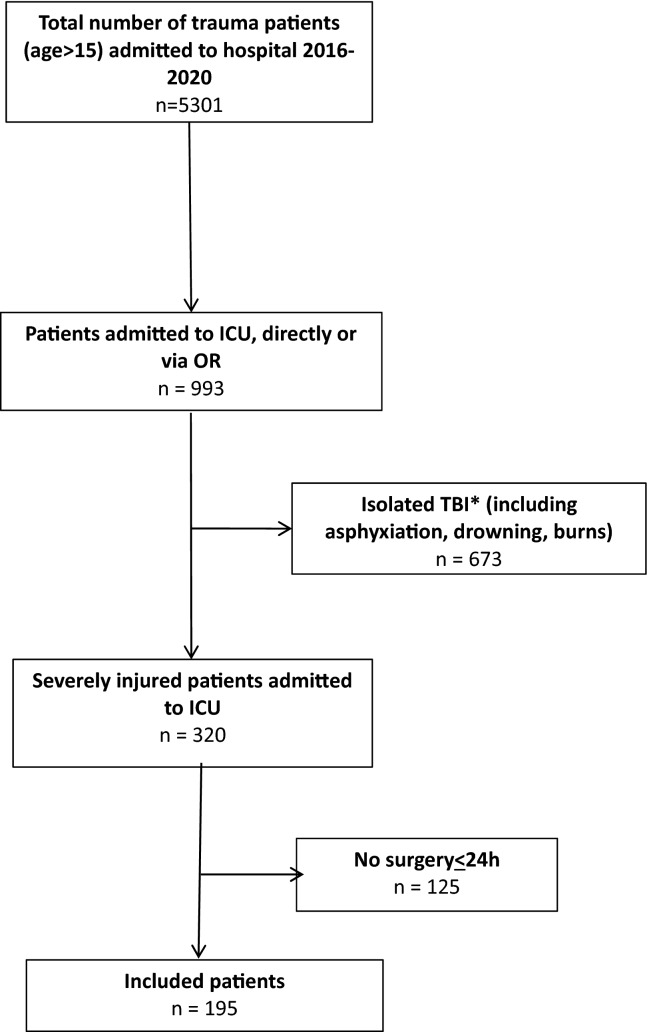
Flowchart of included patients. *Isolated traumatic brain injury (TBI) was defined as Abbreviated Injury Score (AIS) head ≥3 and AIS ≤2 or less in other regions

Hundred ninety-five severely injured patients (66% male) with a median age of 45 (28–60) years who underwent urgent surgery and were admitted to ICU were included. A flowchart of included patients is shown in Fig. 1. Ninety-two percent of injuries (*n* = 179) were caused by a blunt mechanism and median Injury Severity Score (ISS) was 33 (25–38) with most severe injuries located in the brain (Abbreviated Injury Scale (AIS) head 3 (1–4) and chest (AIS chest 3 (1–4)). Physiology, resuscitation and outcome data are presented in Table 1.

**Table 1:** Demographics in polytrauma patients who had urgent surgery comparing patients who underwent damage control surgery (DCS) to patients who had early total care (ETC)

	Total population (*n* = 195)	ETC (*n* = 97)	DCS (*n* = 98)	*P* value
Age (years)	45 (28–60)	49 (33–66)	38 (25–55)	0.009*
Male gender	128 (66)	62 (64)	66 (67)	0.61
Blunt MOI	179 (92)	90 (93)	89 (91)	0.62
ISS	33 (25–38)	33 (26–38)	34 (25–41)	0.70
AIS head	3 (1–4)	3 (2–5)	3 (0–4)	0.006*
AIS face	0 (0–1)	0 (0–2)	0 (0–1)	0.29
AIS chest	3 (1–4)	3 (3–4)	3 (2–4)	0.16
AIS abdomen	2 (0–4)	0 (0–2)	3 (1–4)	< 0.001*
AIS pelvis/extremities	2 (1–3)	2 (0–3)	3 (2–3)	< 0.001*
AIS external	0 (0–1)	0 (0–1)	0 (0–1)	0.51
SBP_ED (mmHg)	116 (90–136)	120 (99–141)	115 (81–130)	0.11
SBP ≤90 mmHg_ED	52 (27)	19 (20)	33 (34)	0.03*
Hb_ED (mmol/L)	7.8 (7.0–8.9)	8.0 (7.2–8.9)	7.6 (6.8–8.9)	0.09
pH_ED	7.31 (7.25–7.37)	7.33 (7.27–7.38)	7.29 (7.23–7.35)	0.005*
PaC02_ED (mmHg)	46 (40–52)	46 (40–52)	46 (40–52)	0.91
BD _ED (mmol/L)	− 3.0 (− 6.0–-1.0)	− 2.0 (− 4.0–0.0)	− 4.0 (− 8.0–− 2.0)	< 0.001*
PT_ED (sec)	14.3 (13.0–15.9)	13.9 (12.5–15.2)	14.7 (13.4–17.3)	0.002*
Temperature_ED (^o^C)	35.5 (34.5–36.5)	35.4 (34.5–36.2)	35.5 (34.6–36.5)	0.88
SBP_ICU (mmHg)	118 (105–137)	121 (109–142)	114 (104–130)	0.03*
Hb_ICU (mmol/L)	7.5 (6.6–8.2)	7.4 (6.6–8.2)	7.6 (6.9–8.2)	0.30
pH_ICU	7.33 (7.28–7.38)	7.35 (7.28–7.39)	7.32 (7.27–7.36)	0.02*
PaCO2_ICU (mmHg)	42 (36–46)	41 (36–47)	42 (36–46)	0.79
BD_ICU (mmol/L)	− 4.3 (− 6.9–− 2.0)	− 3.6 (− 6.2–− 2.0)	− 4.9 (− 7.8–− 2.4)	0.01*
Temperature_ICU (^o^C)	35.4 (34.5–36.0)	35.6 (34.5–36.3)	35.2 (34.3–35.9)	0.18
UO_ICU (ml)	145 (80–300)	140 (80–258)	150 (85–300)	0.71
**Resuscitation parameters**				
Crystalloids ≤24 h (L)	8.7 (6.8–11.0)	8.3 (6.9–10.2)	9.1 (6.7–12.0)	0.06
PRBC ≤24 h (U)	3 (0–7)	2 (0–4)	6 (3–10)	< 0.001*
PRBC ≥10 units ≤24 h	31 (16)	5 (5)	26 (27)	< 0.001*
FFP ≤24 h (U)	3 (0–8)	0 (0–3)	7 (3–12)	< 0.001*
PLT ≤ 24 h (U)^#^	0 (0–1)	0 (0–0)	1 (0–2)	< 0.001*
TXA	158 (81)	68 (70)	90 (92)	< 0.001*
**Outcome parameters**				
Nr of surgeries < 10 days	2 (1–3)	1 (1–2)	3 (2–3)	< 0.001*
Nr of surgeries during H-LOS	2 (1–4)	1 (1–2)	3 (2–4)	< 0.001*
Ventilator days	6 (2–11)	5 (2–11)	7 (2–11)	0.30
Ventilator free days	14 (4–20)	13 (2–20)	15 (5–21)	0.19
ICU LOS (days)	7 (3–14)	6 (3–13)	9 (3–15)	0.36
H-LOS (days)	22 (13–31)	21 (10–29)	23 (15–34)	0.07
MODS	34 (17)	14 (15)	20 (20)	0.27
ARDS	5 (3)	3 (3)	2 (2)	0.64
Infectious complications	93 (48)	42 (44)	51 (52)	0.22
Thrombo-embolic complications	23 (12)	7 (7)	16 (16)	0.05
Mortality	38 (19)	18 (19)	20 (20)	0.74

*MOI* Mechanism of Injury, *ISS* Injury Severity Score, *AIS* Abbreviated Injury Scale, *ED* Emergency Department, *SBP* systolic blood pressure, *Hb* hemoglobin, *PaC02* partial pressure of carbon dioxide in arterial blood, *BD* Base Deficit, *PT* prothrombin time, *UO* urinary output first hr in ICU, *PRBC* packed red blood cells, *FFP* fresh frozen plasma, *PLT* platelets, *TXA* tranexamic acid, *ICU* Intensive Care Unit, *LOS* length of stay, *H-LOS* hospital length of stay, *MODS* Multiple Organ Dysfunction Syndrome, *ARDS* Adult Respiratory Distress Syndrome

Data are expressed in median (IQR) or absolute numbers (%)

*Statistically significant

^#^1 unit of platelets contains five donors

Thirty-eight (19%) patients died; 25 (66%) of them died of TBI, 3 (8%) died of hemorrhage, 3 (8%) died of ischemia after entrapment of the body, 2 (5%) died of respiratory insufficiency, 2 (5%) died of cardiac origin, 1 (3%) due to MODS, 1 (3%) due to hypoxia, and 1(3%) due to sepsis.

### Damage control surgery (DCS) vs early total care (ETC)

Half the patients (*n* = 98) underwent damage control surgery after arrival in hospital. Patients who underwent DCS were younger, more severely injured in regions of the abdomen and pelvis/extremities, and more often in shock with more deranged physiology. They received more blood products ≤ 24 h than patients who did not have DCS. Patients who needed DCS had more surgeries both in the first 10 days and during hospital stay. There was however no difference in outcome between DCS and ETC patients in terms of mortality and non-lethal complications with exception of slightly more thrombo-embolic events in the DCS group (Table 1).

**Fig. 2:**
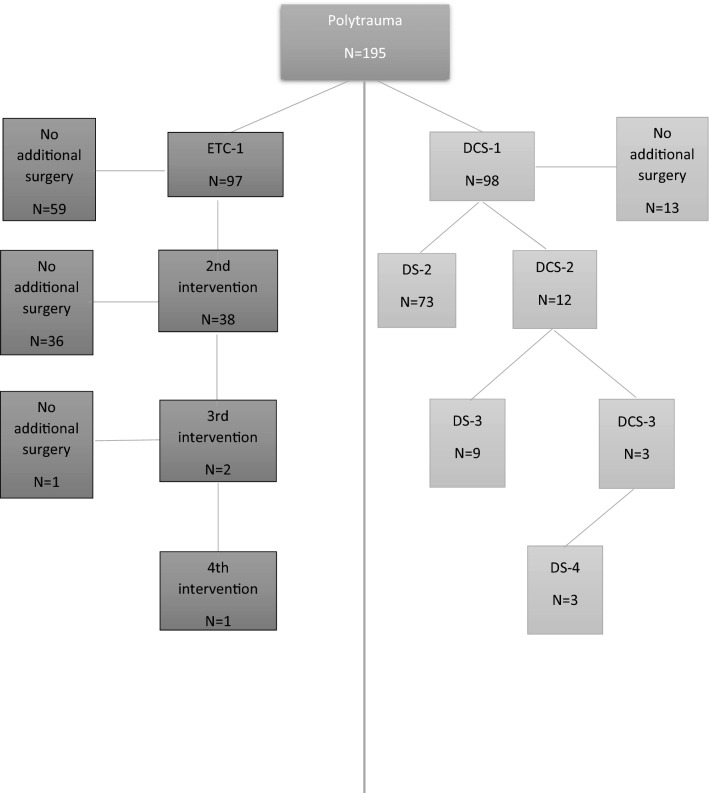
Number of included patients who received damage control surgery (DCS) with subsequent definitive surgery (DS), and/or early total care (ETC)

Figure 2 shows the number of patients who had either DCS or ETC per surgical intervention. Two patients who had ETC in the first surgery had DCS in the second session. The first patient had a vascular injury repaired during the first intervention and underwent a laparotomy and external fixator of the pelvis in the second surgery, with definitive fixation of the pelvis in the third operation. The other patient who had DCS after ETC needed a fasciotomy of an extremity after fracture fixation. The third operation was a damage control laparotomy for abdominal compartment syndrome. The fourth surgery in this particular patient was definitive abdominal closure.

After an initial DCS procedure for resuscitation, 74% of DCS patients (73/98) received definitive surgery (DS) in the second intervention, while 12 patients needed a second damage control procedure (Fig. 2). Eleven of them had a (re)laparotomy, and one had an external fixator on pelvis/extremities. Three of them who had a third DCS surgery, all had a re-laparotomy.

The primary indication to abbreviate surgery was base deficit  ≤-6.0 mmol/L in OR (56%). Other indications were type of injury (multiple (open) long bone fractures, bowel injuries, vertical shear pelvic fractures (21%)), associated severe head injury (16%), and temperature  ≤34 °C in OR (6%, Table 2). Urgent laparotomy (27%) and external fixator of extremities/pelvis (23%) were the most common surgeries performed. All external fixators of extremities/pelvis and 78% of urgent laparotomies were performed in damage control setting, whereas craniotomies and spinal fixations were predominantly performed in early total care setting. Several patients underwent more than one procedure during one session in OR (Table 3). Only four patients had a craniotomy for severe head injury (AIShead  ≥3) combined with damage control surgery for severe injuries in abdomen or pelvis/extremities (AIS abdomen, AIS pelvis/extremities ≥ 3). Two of them died of TBI.

**Table 2:** Indication for damage control surgery (DCS) in first session in OR

Primary indication for DCS	
BD ≤− 6.0 mmol/L_OR	55 (56)
Temperature 34 °C_OR	6 (6)
Associated AIS head ≥3	16 (16)
Type of injuries*	21 (21)
Total	98

*BD* base deficit, *OR* operating room, *AIS* abbreviated injury scale

*Type of injuries: multiple (open) long bone fractures, bowel injury, vertical shear pelvic fracture

**Table 3:** Type of surgery during first session in OR

Type of surgery	ETC (*n* = 97)	DCS (*n* = 98)	Total
Thoracotomy	3 (33)	6 (67)	9 (4)
Laparotomy	15 (22)	53 (78)	68 (27)
Craniotomy	17 (81)	4 (19)	21 (8)
Spine fixation	20 (91)	2 (9)	22 (9)
Fracture fixation	16 (67)	8 (33)	24 (10)
External fixator extremities/pelvis	0	57 (100)	57 (23)
Vascular procedure	11 (61)	7 (39)	18 (7)
Miscellaneous^#^	23 (72)	9 (28)	32 (13)
Total*	105	146	251

Data are expressed as absolute numbers (%)

*Several patients had more than one type of surgery

^#^Miscellaneous procedures included insertion of ICP meter, extraventricular drain, haloframe, amputation extremity, fasciotomy, debridement of soft tissue injuries, neck exploration

When comparing physiological parameters per surgery, DCS patients were more rapidly in OR for their initial procedure (OR-1), and had a more deranged physiology with deeper BD and lower temperature. Further, they received more blood products although there was no difference in the amount of crystalloids during surgery. There was also no difference in hemoglobin levels during this first surgery nor in duration of the procedures (Table 4).

**Table 4:** Physiology and duration of surgery related to damage control surgery (DCS) and subsequent definitive surgery (DS), and early total care (ETC)

OR-1	ETC (*n* = 97)	DCS (*n* = 98)	*P* value
Time from ED to OR (hh:mm)	1:43 (1:02–1:43)	1:01 (0:42–1:20)	< 0.001*
Duration (hh:mm)	2:10 (1:22–3:20)	1:55 (1:30–2:45)	0.51
BD (mmol/L)	− 4.0 (− 6.0–− 1.0)	− 7.0 (− 10.2–− 3.1)	< 0.001*
Hb (mmol/L)	6.8 (5.9–7.8)	6.8 (5.6–7.6)	0.30
Temperature (^o^C)	35.1 (34.5–35.9)	34.8 (33.6–35.4)	0.001*
Crystalloids (L)	3.0 (2.0–5.0)	3.0 (2.0–5.0)	0.76
PRBC (U)	0 (0–2)	3 (1–7)	< 0.001*
FFP (U)	0 (0–2)	4 (1–7)	< 0.001*
PLT (U)^#^	0 (0–0)	0 (0–1)	< 0.001*

OR-2 *	DS (*n* = 73)	DCS (*n* = 12)	
Time from ED to OR (days)	2.0 (1.0–3.0)	0.8 (0.5–1.8)	0.001*
Duration (hh:mm)	3:03 (2:01–4:25)	1:25 (1:10–2:00)	0.002*
BD (mmol/L)	− 1.0 (− 3.0–1.9)	− 3.0 (− 5.2–− 1.3)	0.01*
Hb (mmol/L)	5.7 (4.8–6.4)	6.4 (5.6–7.7)	0.07
Temperature (^o^C)	36.3 (35.5–36.8)	35.9 (35.4–36.3)	0.27
Crystalloids (L)	2.5 (1.6–4.0)	2.0 (1.3–3.0)	0.54
PRBC (U)	1 (0–3)	1 (0–1)	0.66
FFP (U)	0 (0–2)	2 (2–3)	0.03*
PLT (U)^#^	0 0–0)	0 (0–1)	0.14

*ED* Emergency Department, *OR* operating room, *Hb* hemoglobin, *BD* Base Deficit, *PRBC* packed red blood cells, *FFP* fresh frozen plasma, *PLT* platelets

Data are expressed in median (IQR) or absolute numbers (%), * statistically significant

*Only patients who had DCS in OR 1

^#^1 unit of platelets contains five donors

Eighty-five patients (87%) had a second surgery after the first damage control surgery. In this second session in OR (OR-2) DCS took place earlier after admission with shorter duration of the surgical procedure than patients who had definitive surgery (DS). Patients who needed a second DCS had lower base deficit, and received more Fresh Frozen Plasma (FFP). There was however no difference in other parameters (Table 4). Base deficit  ≤-6.0 mmol/L was in only two patients (17%) the indication to abbreviate surgery, in all other patients the type of injuries dictated the abbreviation of the second surgery. All 12 patients who needed 2 damage control procedures in a row had a third operation; 9 (75%) of them had definitive surgery with a median of 2 (1–3) days after admission (DS-3 in Fig. 2). All three patients (25%) who had three damage control procedures in a row had definitive surgery in the fourth session (Fig. 2). No further analysis was performed after the second session in OR since there were too few patients to perform a meaningful analysis.

Seventy-four percent of patients (73/98) who underwent primarily DCS had definitive surgery in the second session in OR (DS-2 in Fig. 2). Median time to this second procedure was 2 (1–3) days.

**Fig. 3:**
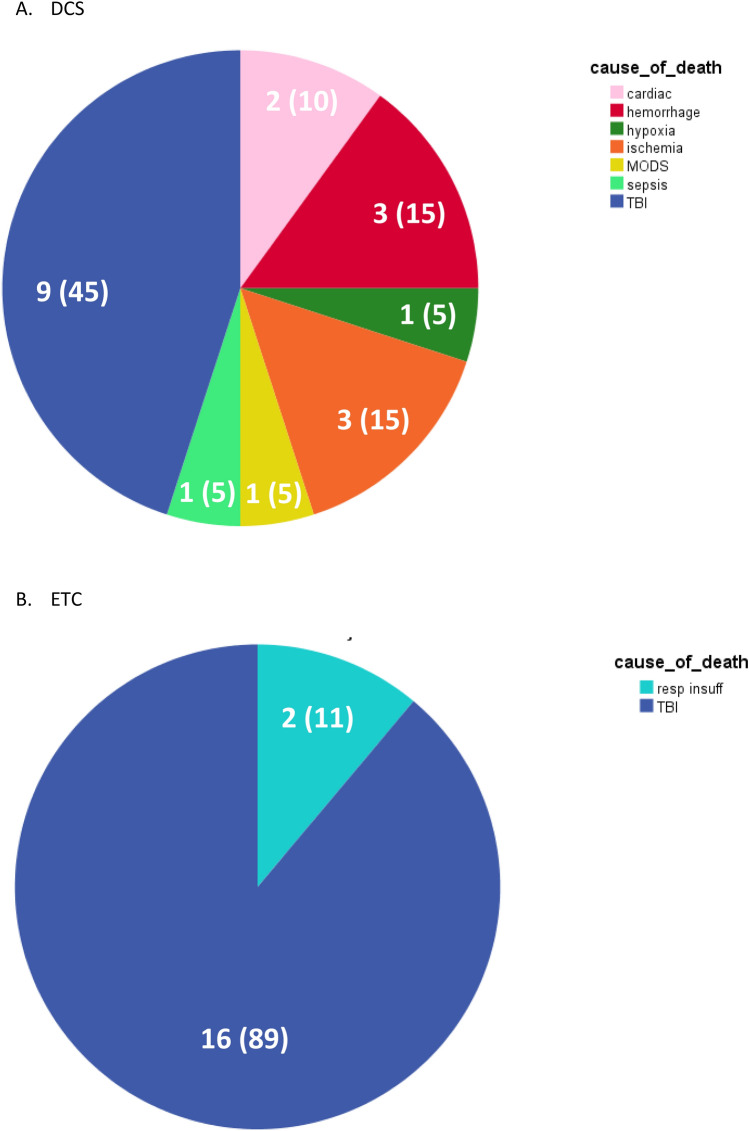
Cause of death in relation to damage control surgery (DCS) or early total care (ETC)*. *Data are expressed as absolute numbers (%)

Although there was no difference in mortality rate between both groups, there was a difference in cause of death: Patients who died after early total care died of TBI in 89% of cases (*n* = 16), the remaining patients (11%, *n* = 2) died of respiratory insufficiency. Although TBI was also the most common cause of death in DCS patients (45%, *n* = 9), there was a wider variety in causes of death including hemorrhage (15%, *n* = 3), ischemia (15%, *n* = 3), sepsis (5%, *n* = 1), cardiac cause (10%, *n* = 2), MODS (5%, *n* = 1), and hypoxia (5%, *n* = 1, Fig. 3).

Thirty-eight patients who needed a second definitive intervention after the first ETC procedure were not treated according to damage control principles by definition, although they received staged surgical (ETC) treatment (Fig. 2). In several of them the separation of the procedures was caused by the fact that the first intervention could technically not be abbreviated, because of the nature of the procedure (craniectomy, endovascular repair for traumatic aortic injury). However, even if these patients were regarded as DCS patients, there was no difference in mortality between DCS and ETC patients (*p* = 0.17).

There was no difference in the development of complications such as MODS, ARDS and infectious complications between DCS and ETC. There was a tendency towards more thrombo-embolic complications in patients who had DCS compared to ETC (16 vs. 7%, *p* = 0.05, Table 1).

In multivariate analysis, BD_ED, AIS head, AIS abdomen, and AIS pelvis/extremities were independent predictors for damage control surgery (Table 5).

**Table 5:** Multivariate analysis: independent predictors for damage control surgery

Variable	β coefficient	*P* value	Odds Ratio	95% C.I	
				Lower	Upper
Age	− 0.015	0.157	0.985	0.965	1.006
pH_ED	0.029	0.257	1.029	0.979	1.081
BD_ED	− 0.017	0.014	0.983	0.970	0.997
SBP ≤90 mmHg_ED	− 0.073	0.890	0.930	0.330	2.617
PT_ED	0.007	0.289	1.007	0.994	1.021
AIS head	− 0.264	0.032	0.768	0.603	0.977
AIS abdomen	0.264	0.034	1.303	1.020	1.663
AIS extr/pelvis	0.725	0.000	2.065	1.499	2.845
Constant	− 23.242	0.218	0.000		

*95% CI* confidence interval, *ED* emergency department, *SBP* systolic blood pressure, *PT* prothrombin time, *AIS* abbreviated injury scale

## Discussion

In this cohort of polytrauma patients who underwent urgent surgery, there was no difference in outcome in terms of mortality and non-lethal complications between patients who received DCS and those who did not, even though DCS patients had a more deranged physiology and needed more resuscitation.

When analyzing separate OR procedures more into detail, DCS patients had a more deranged physiology than ETC patients during the first session. Interestingly, this difference in physiology faded during second surgery. DCS patients who needed a second DCS procedure had this done within 24 h after admission. Further, all patients who initially needed one or two DCS procedures had definitive surgery 2 days after injury. This suggests that resuscitation in DCS group was prompt and adequate, and that 2 days after injury seemed to be a safe time point to start definitive surgery. This is in contrast with others reporting that postinjury days 2 to 4 are not ideal to perform secondary definitive operations because of ongoing inflammatory response [10]. The reason for these differences could possibly be due to a fairly low threshold for damage control surgery; Only 34% of DCS patients had SBP  ≤90 mmHg on arrival in ED, and less than one-third received massive transfusion in the first 24 h. This could be partly explained by a phenomenon previously described in which severely injured patients in smaller service areas with short transport times do not have severely deranged commonly used physiologic parameters on arrival in ED, because they do not have the time to fully deteriorate prior to arrival in hospital [14, 21, 22]. Although base deficit in ED was only − 4.0 mmol/L in DCS patients, it decreased to − 7.0 mmol/L during the first surgery compared to a BD drop from − 2.0 to − 4.0 mmol/L in ETC patients. Additionally, during the second intervention, DCS patients who went back to OR more quickly with shorter OR times still had a more deranged base deficit than ETC patients. This demonstrates that physiology was still dictating treatment during the second period in OR. It also shows that patient selection for abbreviated surgery, based on anatomy and injury severity in combination with the physiological derangement and anticipated deterioration, was correct.

Data also demonstrate that resuscitation with blood products in DCS patients during surgery was adequate since hemoglobin level was similar between both groups during the first surgery even though DCS patients received more blood products. Another reason for similar hemoglobin levels could be the fact that surgery was not only abbreviated due to hemorrhage but also because of other reasons such as associated TBI.

Base deficit in ED was the most important independent physiological predictor for the decision to perform damage control surgery. All other independent predictors were based on injury type.

As could be expected, the type of surgery between DCS and ETC was different with external fixators exclusively being used in DCS, and spine fixation and craniotomies mainly performed in ETC. Seventy-eight percent of laparotomies were performed in a damage control setting. This is comparable to many other Level-1 trauma centers studied by Watson et al. [4].

Although there was no difference in ISS between DCS and ETC, there was a difference in injury pattern; ETC patients had more severe head injuries with more craniotomies, whereas patients with more severely injuries in abdomen and pelvis/extremities underwent more often abbreviated surgery. These differences in type of injuries between DCS and ETC patients was reflected by difference in cause of death. Further, none of the ETC patients died of hemorrhage suggesting the selection to perform DCS was accurate.

Only four patients (2%) underwent a craniotomy for severe head injury combined with damage control surgery for severe abdominal or pelvis/extremity injuries. These low numbers are likely due to the fact that this combination of severe injuries is often fatal prior to arrival in hospital.

There was no difference in complications between DCS and ETC patients even though DCS patients had more surgeries both within the first 10 days and during their stay in hospital. However, there was a tendency towards more thrombo-embolic events in the DCS group though numbers were low. This is in contrast with several other studies reporting more complications after damage control [6–9].

Timing of surgery remains an ongoing debate and many strategies for treating polytrauma patients are described in literature with a recent interest in EAC [13]. The question is whether there is a clinically relevant difference between EAC and ETC. Eventually, the most important factor is that patients who need abbreviated surgery are correctly selected. The decision for (abbreviated) surgery in this study was based on several factors including physiological parameters, anatomical locations of the injuries, associated injuries, patient’s response to the given care, and surgeon’s discretion. In our opinion, managing polytrauma patients requires a tailor-made approach for the individual patient. Since one of the goals of this study was to evaluate the decision to abbreviate surgery, we decided to use the historical terms of DCS and ETC to avoid any confusion.

To our knowledge, this is the first study in which both damage control surgery in truncal injuries and fractures are compared to early total care per surgical intervention. Data demonstrated that the decision for DCS based on physiological derangement in combination with anatomical location and severity of the injuries was prompt and adequate. DCS patients had similar outcome compared to ETC patients despite having a more deranged physiology in the early phase after trauma.

A few limitations need to be acknowledged: First, this was a retrospective analysis of a single center prospective cohort study with its accompanying limits. Further, treating clinicians were also the researchers. Another limitation is that no details on comorbidities were collected nor any long-term complications such as enterocutaneous fistulas or ventral hernias.

In conclusion, when in severely injured patients treatment is dictated by physiology into either early definitive surgery or damage control with multiple shorter procedures stretched over several days combined with aggressive resuscitation with blood products, outcome is comparable in terms of complications such as mortality, organ failure and infections.

## Supplementary Information

Below is the link to the electronic supplementary material.

Supplementary file1 (DOCX 30 KB)

## Data Availability

The dataset supporting the conclusions of this article are available upon reasonable request from the corresponding author.
